# 5-Chloro-*N*-[2-(1*H*-imidazol-4-yl)eth­yl]-*N*-methyl-7*H*-pyrrolo[2,3-*d*]pyrimidin-4-amine

**DOI:** 10.1107/S1600536809054750

**Published:** 2009-12-24

**Authors:** Daniel Richter, John C. Kath, Arnold L. Rheingold, Antonio DiPasquale, Alex Yanovsky

**Affiliations:** a Pfizer Global Research and Development, La Jolla Labs, 10770 Science Center Drive, San Diego, CA 92121, USA; b Department of Chemistry and Biochemistry, University of California, San Diego, 9500 Gilman Drive, La Jolla, CA 92093, USA

## Abstract

The title compound, C_12_H_13_ClN_6_, was prepared by reaction of 4,5-dichloro-7*H*-pyrrolo[2,3-*d*]pyrimidine with 2-(1*H*-imid­azol-4-yl)-*N*-methyl­ethanamine, and the X-ray study confirmed that chloro-substituent in six-membered ring was replaced in the reaction. The exocyclic N atom environment is approximately coplanar with the pyrrolo[2,3-*d*]pyrimidine [corresponding dihedral angle is 5.5 (1)°], whereas the mean plane of the N—C—C—C link connecting with the imidazolyl ring is almost exactly orthogonal to the plane of the bicyclic system [dihedral angle = 91.6 (2)°]. The imidazolyl plane itself, however, forms a relatively small dihedral angle of 20.8 (1)° with the pyrrolo[2,3-*d*]pyrimidine plane. There are two independent N—H⋯N hydrogen bonds in the structure, which link mol­ecules into layers parallel to (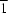
03).

## Related literature

For the structures of related compounds with the pyrrolo[2,3-*d*]pyrimidin-4-amine bicyclic framework, see: Abola & Sundaralingam (1973 ▶); Slauson *et al.* (2008 ▶); Zabel *et al.* (1987 ▶).
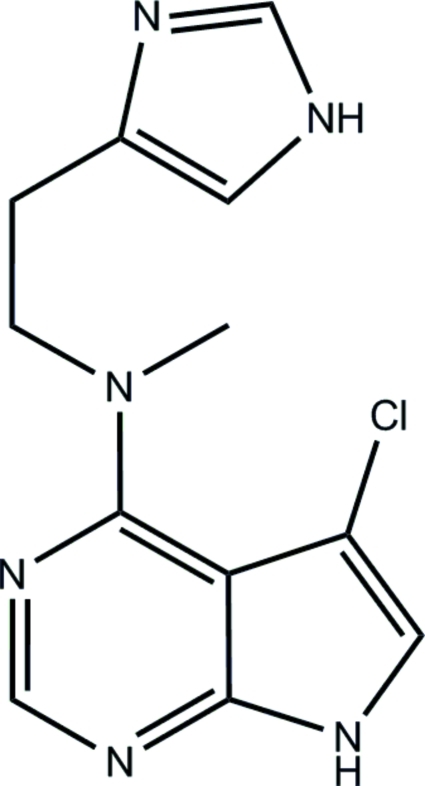


## Experimental

### 

#### Crystal data

C_12_H_13_ClN_6_
*M* *_r_* = 276.73
Monoclinic, 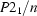

*a* = 4.4673 (5) Å
*b* = 15.8855 (17) Å
*c* = 17.6544 (19) Å
β = 96.244 (2)°
*V* = 1245.4 (2) Å^3^
*Z* = 4
Mo *K*α radiation
μ = 0.30 mm^−1^
*T* = 208 K
0.16 × 0.08 × 0.08 mm

#### Data collection

Bruker SMART CCD area-detector diffractometer
Absorption correction: multi-scan (*SADABS*; Bruker, 2001 ▶) *T*_min_ = 0.953, *T*_max_ = 0.976
8932 measured reflections
2669 independent reflections
2223 reflections with *I* > 2σ(*I*)
*R*_int_ = 0.029

#### Refinement

*R*[*F* ^2^ > 2σ(*F* ^2^)] = 0.043
*wR*(*F* ^2^) = 0.123
*S* = 1.05
2669 reflections
173 parameters
H-atom parameters constrained
Δρ_max_ = 0.38 e Å^−3^
Δρ_min_ = −0.55 e Å^−3^

### 

Data collection: *SMART* (Bruker, 1997 ▶); cell refinement: *SAINT* (Bruker, 1997 ▶); data reduction: *SAINT*; program(s) used to solve structure: *SIR97* (Burla *et al.*, 2005 ▶); program(s) used to refine structure: *SHELXL97* (Sheldrick, 2008 ▶); molecular graphics: *ORTEP-32* (Farrugia, 1997 ▶) and *PLATON* (Spek, 2009 ▶); software used to prepare material for publication: *WinGX* (Farrugia, 1999 ▶).

## Supplementary Material

Crystal structure: contains datablocks global, I. DOI: 10.1107/S1600536809054750/dn2525sup1.cif

Structure factors: contains datablocks I. DOI: 10.1107/S1600536809054750/dn2525Isup2.hkl

Additional supplementary materials:  crystallographic information; 3D view; checkCIF report

## Figures and Tables

**Table 1:** Hydrogen-bond geometry (Å, °)

*D*—H⋯*A*	*D*—H	H⋯*A*	*D*⋯*A*	*D*—H⋯*A*
N2—H2⋯N5^i^	0.87	1.98	2.845 (2)	175
N6—H6*A*⋯N3^ii^	0.87	2.04	2.892 (2)	167

Symmetry codes: (i) 
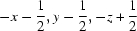
; (ii) 
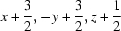
.

**Fig. 1.**
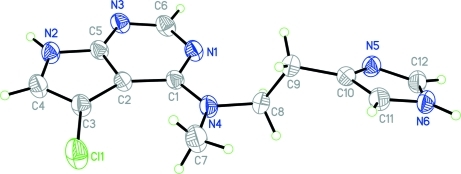
Molecular structure of the title compound, showing 50% probability displacement ellipsoids and atom numbering scheme. H atoms are drawn as circles with arbitrary small radius.

**Fig. 2.**
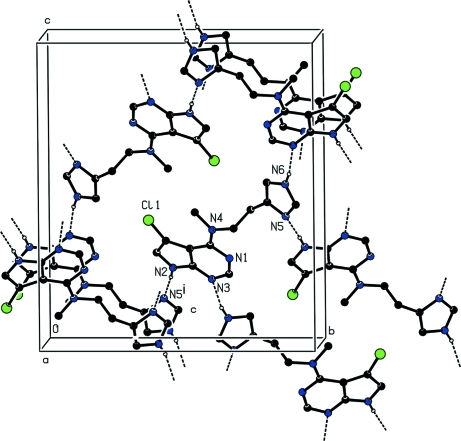
Partial packing view of the title compound, viewed down the a axis. Hydrogen bonds are shown as dashed lines. H atoms not involved in hydrogen bondings have been omitted for clarity. [Symmetry code: (i) -x-1/2, y-1/2, -z+1/2]

## supplementary crystallographic information

### Comment

The title compound, C_12_H_13_ClN_6_, was prepared by reaction of 4,5-dichloro-7*H*-pyrrolo[2,3-*d*]pyrimidine with 2-(1*H*-imidazol-4-yl)-*N*-methylethanamine, and the present X-ray study confirmed that chloro-substituent in six-membered ring got replaced in this reaction (Fig. 1).

The pyrrolo[2,3-*d*]pyrimidine system is planar within 0.001 Å, and its least-squares plane, C1/C2/C3/C4/N2/C5/N3/C6/N1 is almost coplanar with the plane C1/N4/C7/C8, the corresponding dihedral angle being equal to 5.5 (1)° (the maximum deviation of the N4 atom from the latter plane being 0.028 (2) Å). The N4—C8—C9—C10 chain linking the bicyclic system with the imidazolyl group may be considered as approximately planar (within 0.130 Å) and its mean plane is orthogonal to the plane of pyrrolopyrimidine [91.6 (2)°]. At the same time the imidazolyl plane forms a relatively small dihedral angle of 20.8 (1)° with the bicyclic system.

The geometric parameters of pyrrolopyrimidin-4-amine system are similar to those observed in related structures (Zabel *et al.*, 1987; Abola & Sundaralingam, 1973), although the title compound provides the first structure with no substitution at the N atom in the pyrrole part. The only other structurally studied compound with disubstituted 4-amino-group (Slauson *et al.*, 2008) shows noticeable non-planarity of the environment of the exocyclic N atom.

There are two independent H-bonds in the structure (Table 1) which link molecules into layers parallel to (-1,0,3) plane (Fig. 2).

### Experimental

To a mixture of 4,5-dichloro-7*H*-pyrrolo[2,3-*d*]pyrimidine (119 mg, 0.63 mmol) and [2-(1*H*-imidazol-4-yl)-ethyl]-methylamine (79 mg, 0.63 mmol) dissolved in 2-propanol (1 ml) was added di-isopropyl-ethylamine (0.12 ml, 1.1 eq). The reaction was heated at 80°C for 18 hrs. The solvent was removed and the reaction purified on Si—NH_2_ (6% MeOH/EtOAc); product was isolated as colorless solid, 48 mg (27%). 1H NMR (400 MHz, DMSO-d6) δ p.p.m. 2.85 - 2.91 (m, 2 H), 3.22 (s, 3 H), 3.88 (dd, J=8.56, 6.80 Hz, 2 H), 6.78 (s, 1 H), 7.41 (d, J=1.76 Hz, 1 H), 7.52 (d, J=1.01 Hz, 1 H), 8.17 (s, 1 H), 12.05 (s, 1 H).

### Refinement

All H atoms were placed in geometrically calculated positions (C—H 0.97 Å for methyl, 0.98 Å for methylene, 0.94 Å for aromatic CH-groups; N—H 0.87 Å) and included in the refinement in riding motion approximation. The *U*_iso_(H) were set to 1.2*U*_eq_ of the carrying atom [1.5*U*_eq_ for methyl H atoms].

### Crystal data

**Table:**

C_12_H_13_ClN_6_	*F*(000) = 576
*M**_r_* = 276.73	*D*_x_ = 1.476 Mg m^−^^3^
Monoclinic, *P*2_1_/*n*	Mo *K*α radiation, λ = 0.71073 Å
Hall symbol: -P 2yn	Cell parameters from 4149 reflections
*a* = 4.4673 (5) Å	θ = 2.3–27.6°
*b* = 15.8855 (17) Å	µ = 0.30 mm^−^^1^
*c* = 17.6544 (19) Å	*T* = 208 K
β = 96.244 (2)°	Rod, colorless
*V* = 1245.4 (2) Å^3^	0.16 × 0.08 × 0.08 mm
*Z* = 4	

### Data collection

**Table:**

Bruker SMART CCD area-detector diffractometer	2669 independent reflections
Radiation source: fine-focus sealed tube	2223 reflections with *I* > 2σ(*I*)
graphite	*R*_int_ = 0.029
phi and ω scans	θ_max_ = 27.7°, θ_min_ = 1.7°
Absorption correction: multi-scan (*SADABS*; Bruker, 2001)	*h* = −5→2
*T*_min_ = 0.953, *T*_max_ = 0.976	*k* = −20→19
8932 measured reflections	*l* = −22→22

### Refinement

**Table:**

Refinement on *F*^2^	Primary atom site location: structure-invariant direct methods
Least-squares matrix: full	Secondary atom site location: difference Fourier map
*R*[*F*^2^ > 2σ(*F*^2^)] = 0.043	Hydrogen site location: inferred from neighbouring sites
*wR*(*F*^2^) = 0.123	H-atom parameters constrained
*S* = 1.05	*w* = 1/[σ^2^(*F*_o_^2^) + (0.0625*P*)^2^ + 0.5128*P*] where *P* = (*F*_o_^2^ + 2*F*_c_^2^)/3
2669 reflections	(Δ/σ)_max_ = 0.001
173 parameters	Δρ_max_ = 0.38 e Å^−^^3^
0 restraints	Δρ_min_ = −0.55 e Å^−^^3^

### Special details

**Table:**

Geometry. All e.s.d.'s (except the e.s.d. in the dihedral angle between two l.s. planes) are estimated using the full covariance matrix. The cell e.s.d.'s are taken into account individually in the estimation of e.s.d.'s in distances, angles and torsion angles; correlations between e.s.d.'s in cell parameters are only used when they are defined by crystal symmetry. An approximate (isotropic) treatment of cell e.s.d.'s is used for estimating e.s.d.'s involving l.s. planes.
Refinement. Refinement of *F*^2^ against ALL reflections. The weighted *R*-factor *wR* and goodness of fit *S* are based on *F*^2^, conventional *R*-factors *R* are based on *F*, with *F* set to zero for negative *F*^2^. The threshold expression of *F*^2^ > σ(*F*^2^) is used only for calculating *R*-factors(gt) *etc*. and is not relevant to the choice of reflections for refinement. *R*-factors based on *F*^2^ are statistically about twice as large as those based on *F*, and *R*- factors based on ALL data will be even larger.

### Fractional atomic coordinates and isotropic or equivalent isotropic displacement parameters (Å^2^)

**Table:**

	*x*	*y*	*z*	*U*_iso_*/*U*_eq_	
C1	−0.0192 (4)	0.58232 (11)	0.29931 (10)	0.0283 (4)	
C2	−0.1622 (4)	0.50275 (10)	0.28217 (10)	0.0277 (4)	
C3	−0.1729 (4)	0.41555 (11)	0.30729 (11)	0.0328 (4)	
C4	−0.3817 (4)	0.37368 (12)	0.25972 (11)	0.0358 (4)	
H4	−0.4315	0.3165	0.2638	0.043*	
C5	−0.3795 (4)	0.50431 (11)	0.21665 (10)	0.0297 (4)	
C6	−0.2998 (4)	0.63935 (11)	0.19349 (11)	0.0351 (4)	
H6	−0.3424	0.6876	0.1634	0.042*	
C7	0.2759 (5)	0.53923 (14)	0.41905 (14)	0.0525 (6)	
H7A	0.0959	0.5146	0.4359	0.079*	
H7B	0.3892	0.5680	0.4614	0.079*	
H7C	0.3992	0.4952	0.4004	0.079*	
C8	0.3331 (4)	0.68204 (12)	0.36897 (11)	0.0346 (4)	
H8A	0.3273	0.7100	0.3194	0.042*	
H8B	0.5448	0.6750	0.3891	0.042*	
C9	0.1773 (4)	0.73809 (12)	0.42332 (11)	0.0339 (4)	
H9A	−0.0034	0.7630	0.3957	0.041*	
H9B	0.1139	0.7035	0.4647	0.041*	
C10	0.3803 (4)	0.80697 (11)	0.45646 (10)	0.0297 (4)	
C11	0.5339 (4)	0.81184 (12)	0.52734 (11)	0.0344 (4)	
H11	0.5279	0.7730	0.5673	0.041*	
C12	0.6424 (4)	0.92068 (12)	0.45984 (11)	0.0354 (4)	
H12	0.7291	0.9715	0.4459	0.042*	
N1	−0.0952 (3)	0.64914 (9)	0.25352 (9)	0.0331 (3)	
N2	−0.5079 (3)	0.42744 (9)	0.20513 (9)	0.0336 (3)	
H2	−0.6479	0.4145	0.1688	0.040*	
N3	−0.4509 (3)	0.57078 (10)	0.17064 (9)	0.0340 (3)	
N4	0.1919 (3)	0.59882 (10)	0.35853 (9)	0.0351 (4)	
N5	0.4505 (3)	0.87609 (10)	0.41396 (9)	0.0325 (3)	
N6	0.6992 (3)	0.88485 (10)	0.52872 (9)	0.0357 (4)	
H6A	0.8182	0.9042	0.5670	0.043*	
Cl1	0.02168 (14)	0.36102 (3)	0.38240 (3)	0.0529 (2)	

### Atomic displacement parameters (Å^2^)

**Table:**

	*U*^11^	*U*^22^	*U*^33^	*U*^12^	*U*^13^	*U*^23^
C1	0.0312 (8)	0.0258 (8)	0.0273 (9)	0.0014 (6)	0.0001 (6)	−0.0027 (7)
C2	0.0335 (8)	0.0243 (8)	0.0244 (9)	0.0013 (6)	−0.0012 (6)	−0.0004 (6)
C3	0.0414 (9)	0.0262 (9)	0.0291 (9)	−0.0002 (7)	−0.0047 (7)	0.0041 (7)
C4	0.0457 (10)	0.0258 (9)	0.0344 (10)	−0.0026 (7)	−0.0026 (7)	0.0009 (7)
C5	0.0339 (8)	0.0264 (9)	0.0277 (9)	0.0022 (7)	−0.0018 (6)	−0.0023 (7)
C6	0.0470 (10)	0.0275 (9)	0.0297 (10)	0.0045 (7)	−0.0016 (7)	0.0042 (7)
C7	0.0680 (13)	0.0322 (11)	0.0498 (14)	0.0004 (10)	−0.0272 (10)	0.0014 (9)
C8	0.0321 (8)	0.0318 (9)	0.0389 (10)	−0.0054 (7)	−0.0013 (7)	−0.0069 (8)
C9	0.0321 (8)	0.0321 (9)	0.0366 (10)	−0.0037 (7)	−0.0003 (7)	−0.0046 (8)
C10	0.0294 (7)	0.0277 (9)	0.0311 (9)	0.0024 (6)	−0.0005 (6)	−0.0029 (7)
C11	0.0395 (9)	0.0298 (9)	0.0323 (10)	0.0011 (7)	−0.0025 (7)	0.0004 (7)
C12	0.0386 (9)	0.0283 (9)	0.0376 (11)	−0.0030 (7)	−0.0036 (7)	−0.0024 (8)
N1	0.0408 (8)	0.0250 (7)	0.0327 (9)	0.0002 (6)	0.0004 (6)	−0.0001 (6)
N2	0.0387 (7)	0.0285 (8)	0.0312 (8)	−0.0022 (6)	−0.0069 (6)	−0.0021 (6)
N3	0.0415 (8)	0.0282 (8)	0.0301 (8)	0.0048 (6)	−0.0060 (6)	0.0008 (6)
N4	0.0407 (8)	0.0257 (8)	0.0359 (9)	−0.0009 (6)	−0.0090 (6)	−0.0031 (6)
N5	0.0349 (7)	0.0303 (8)	0.0305 (8)	−0.0009 (6)	−0.0048 (6)	−0.0002 (6)
N6	0.0390 (8)	0.0320 (8)	0.0331 (9)	−0.0011 (6)	−0.0094 (6)	−0.0062 (7)
Cl1	0.0763 (4)	0.0314 (3)	0.0444 (3)	−0.0069 (2)	−0.0233 (3)	0.0115 (2)

### Geometric parameters (Å, °)

**Table:**

C1—N1	1.355 (2)	C7—H7C	0.9700
C1—N4	1.355 (2)	C8—N4	1.468 (2)
C1—C2	1.434 (2)	C8—C9	1.531 (3)
C2—C5	1.427 (2)	C8—H8A	0.9800
C2—C3	1.457 (2)	C8—H8B	0.9800
C3—C4	1.359 (3)	C9—C10	1.499 (2)
C3—Cl1	1.7360 (18)	C9—H9A	0.9800
C4—N2	1.363 (2)	C9—H9B	0.9800
C4—H4	0.9400	C10—C11	1.362 (2)
C5—N3	1.349 (2)	C10—N5	1.385 (2)
C5—N2	1.355 (2)	C11—N6	1.374 (2)
C6—N3	1.321 (2)	C11—H11	0.9400
C6—N1	1.331 (2)	C12—N5	1.320 (2)
C6—H6	0.9400	C12—N6	1.342 (2)
C7—N4	1.446 (3)	C12—H12	0.9400
C7—H7A	0.9700	N2—H2	0.8700
C7—H7B	0.9700	N6—H6A	0.8700
			
N1—C1—N4	114.64 (15)	H8A—C8—H8B	107.8
N1—C1—C2	119.20 (15)	C10—C9—C8	111.87 (14)
N4—C1—C2	126.15 (16)	C10—C9—H9A	109.2
C5—C2—C1	113.83 (15)	C8—C9—H9A	109.2
C5—C2—C3	102.77 (14)	C10—C9—H9B	109.2
C1—C2—C3	143.40 (16)	C8—C9—H9B	109.2
C4—C3—C2	108.69 (15)	H9A—C9—H9B	107.9
C4—C3—Cl1	118.73 (14)	C11—C10—N5	109.35 (16)
C2—C3—Cl1	132.58 (14)	C11—C10—C9	128.54 (17)
C3—C4—N2	109.48 (16)	N5—C10—C9	122.03 (16)
C3—C4—H4	125.3	C10—C11—N6	106.29 (16)
N2—C4—H4	125.3	C10—C11—H11	126.9
N3—C5—N2	123.13 (15)	N6—C11—H11	126.9
N3—C5—C2	126.66 (16)	N5—C12—N6	112.00 (17)
N2—C5—C2	110.21 (15)	N5—C12—H12	124.0
N3—C6—N1	128.55 (17)	N6—C12—H12	124.0
N3—C6—H6	115.7	C6—N1—C1	119.29 (15)
N1—C6—H6	115.7	C5—N2—C4	108.84 (15)
N4—C7—H7A	109.5	C5—N2—H2	125.6
N4—C7—H7B	109.5	C4—N2—H2	125.6
H7A—C7—H7B	109.5	C6—N3—C5	112.45 (15)
N4—C7—H7C	109.5	C1—N4—C7	123.13 (16)
H7A—C7—H7C	109.5	C1—N4—C8	121.64 (15)
H7B—C7—H7C	109.5	C7—N4—C8	115.03 (15)
N4—C8—C9	112.60 (15)	C12—N5—C10	105.27 (16)
N4—C8—H8A	109.1	C12—N6—C11	107.09 (15)
C9—C8—H8A	109.1	C12—N6—H6A	126.5
N4—C8—H8B	109.1	C11—N6—H6A	126.5
C9—C8—H8B	109.1		

### Hydrogen-bond geometry (Å, °)

**Table:**

*D*—H···*A*	*D*—H	H···*A*	*D*···*A*	*D*—H···*A*
N2—H2···N5^i^	0.87	1.98	2.845 (2)	175
N6—H6A···N3^ii^	0.87	2.04	2.892 (2)	167

Symmetry codes: (i) −*x*−1/2, *y*−1/2, −*z*+1/2; (ii) *x*+3/2, −*y*+3/2, *z*+1/2.
